# Yttrium-90 radioembolization for neuroendocrine liver metastases: baseline vascularity predicts cytoreduction efficacy

**DOI:** 10.1371/journal.pone.0352018

**Published:** 2026-07-14

**Authors:** Anne Frisch, Johannes Kahn, Federico Collettini, Peter Steinhagen, Uli Fehrenbach, Bernhard Gebauer, Holger Amthauer, Imke Schatka, Paul Herzler, Gero Wieners, Willie M. Lüdemann

**Affiliations:** 1 Department of Diagnostic and Interventional Radiology, Medizinische Fakultät Ostwestfalen Lippe, Bielefeld, Germany; 2 Department of Diagnostic and Interventional Radiology, Health and Medical University Potsdam, Potsdam, Germany; 3 Department of Radiology, Charité-Universitätsmedizin Berlin, Corporate Member of Freie Universität Berlin and Humboldt-Universität zu Berlin, Berlin, Germany; 4 Department of Hepatology and Gastroenterology, Charité-Universitätsmedizin Berlin, Corporate Member of Freie Universität Berlin and Humboldt-Universität zu Berlin, Berlin, Germany; 5 Department of Nuclear Medicine, Charité-Universitätsmedizin Berlin, Corporate Member of Freie Universität Berlin and Humboldt-Universität zu Berlin, Berlin, Germany; 6 Department of Radiology, Medizinische Universität Lausitz – Carl Thiem, Cottbus, Germany; Fondazione Policlinico Universitario Agostino Gemelli IRCCS, ITALY

## Abstract

**Purpose:**

This retrospective study in patients with neuroendocrine liver metastases analyzes baseline vascularity on MR as a predictor of tumour debulking after conventional Yttrium-90 transarterial radioembolization (TARE).

**Methods:**

30 patients who underwent TARE at our center between 2015 and 2022 were retrospectively assessed. Pre- and post-treatment target lesion volumes were measured as total tumor volume (TTV) and enhancing tumor volume (ETV). Cox-regression analyses as well as Kaplan-Meier statistics were used to evaluate baseline volumetric imaging parameters and clinical variables as predictors of survival.

**Results:**

Mean patients’ age was 63 (SD 11) years, 15 were female (50%). The primary tumor was in the upper gastrointestinal tract in 13/30 cases (43%). Median overall survival (mOS) was 33.0 months (95%CI [21.2; 44.7]). Eligibility for peptide receptor radionuclide therapy (PRRT) was the single strongest predictor of survival with a hazard ratio of 0.22 (p = 0.024) and a mOS of 60.8 months whereas receptor-negative patients had the shortest survival of 7.0 months (p = 0.019). A baseline ETV/TTV ratio greater 50% was associated with a decrease in median ETV by 41% after lobar TARE compared to an increase by 18% in patients with poor baseline vascularity (p = 0.018). In the 17/30 patient who received systemic therapies exclusively before TARE, mOS was 15 vs. 6.8 months (p = 0.082). Prior PRRT did not significantly impact the change of ETV after TARE.

**Conclusion:**

In lobar TARE for neuroendocrine liver metastases, high baseline vascularity shows an exploratory association with a significant reduction in viable tumor volume, warranting further investigation to improve patient selection.

## Purpose

Transarterial Yttrium-90 (Y-90) radioembolization (TARE) is a minimally invasive intraarterial therapy for unresectable both primary and secondary liver tumors such as hepatocellular carcinoma (HCC), colorectal liver metastases (CRLM) or, less prevalent, neuroendocrine liver metastases (NELM) [1]. Predictors of local response are subject of intense research to improve patient selection. In this regard, the role of baseline lesion vascularity merits further investigation.

Neuroendocrine neoplasms are rare malignancies with a prevalence of 35 per 100,000 in the United States [2]. Due to the frequent lack of early symptoms, distant metastases predominantly to the liver are seen in up to 27% of all patients at initial presentation and up to 80% will develop liver metastases at a later stage [3,4]. Liver metastases negatively impact quality of life due to bulk symptoms or carcinoid syndromes and are associated with decreased patient survival [5]. As complete resection of NELMs is feasible in only 20% of patients, systemic therapies with somatostatin analogues, peptide receptor radionuclide therapy (PRRT) or chemotherapy, liver transplantation in highly selected patients, cytoreductive surgical or ablative techniques and transarterial liver-directed-therapies (LDTs) are used to manage these patients [5–7]. With respect to transarterial LDTs, comparative studies demonstrate similar efficacy of transarterial embolization (TAE) or chemoembolization (TACE) and TARE for disease control whereas high-quality data is still lacking [3]. As long-term liver toxicity following TARE has been observed, the use of TAE and TACE rather than TARE has been advocated in the era of PRRT. In patients with colonized biliary systems however, TARE is generally preferred over other embolotherapies [8,9]. During TARE, radionuclides such as Yttrium-90 or Holmium-166 embedded in microspheres are applied via branches of the hepatic artery. It can be performed as lobar therapy in disseminated disease or selectively as radiation segmentectomy minimizing radiation to non-affected liver tissue [10]. Given the predominantly arterial blood supply of hypervascularized tumors, the microspheres accumulate in the tumor micro-vasculature and emit high-energy, low-penetration beta radiation to the tumor [11]. The DOSISPHERE trial demonstrated that conventional body surface area (BSA)–based dosimetry, as employed in earlier studies, may result in insufficient tumour doses, leading to lower response and survival rates compared with personalized dosimetry, which represents the current standard of care based on Technetium-99m macroaggregated albumin SPECT/CT [12,13]. Recent evidence in TARE for CRLM, HCC, and NELM suggests a strong association between local treatment response and patient survival, as well as baseline metabolic or perfusion-related tumour characteristics, such as relative vascularization on MRI, apparent diffusion coefficient, or tracer uptake on positron emission tomography [14–16]. A recent analysis in HCC who underwent conventional lobar TARE demonstrated a baseline relative arterial vascularity greater 50% to be associated with a consistent reduction in viable tumour volume, prolonged time to target lesion progression and potential survival benefits in combination with systemic therapy, compared to patients with low baseline vascularity [17].

The primary aim of this retrospective study is to evaluate whether baseline vascularity cutoffs in NELMs can improve the prediction of treatment response following conventional TARE. Secondary outcomes include exploratory analyses of potential clinical implications, incorporating survival data and relevant clinical confounders.

## Methods

### Study cohort

**Fig 1:**
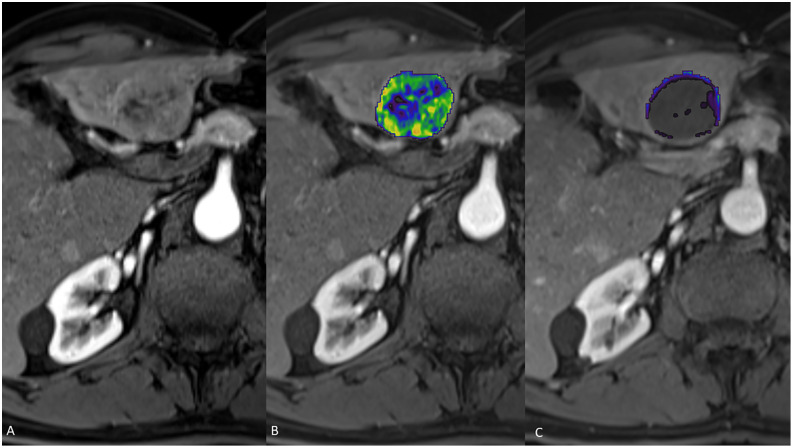
Imaging example. A: A neuroendocrine liver metastasis in the left lobe is shown in the arterial MR contrast phase. B: The lesion is segmented; arterial vascularization is defined as an increase in intensity greater two standard deviations than the average signal intensity measured within a region of interest (ROI) of 10 x 10 x 10 mm in the musculus erector spinae (outside the imaging plane). Relative vascularization is calculated as ratio of enhancing and total tumour volume (ETV/TTV), in this example exceeding 50%. C: This is the same lesion three months after treatment with typical residual rim enhancement.

This retrospective single-institution study was approved by the institutional ethics committee. Informed consent was waived due to the retrospective design of the study. All procedures performed in studies involving human participants were in accordance with the ethical standards of the institutional and/or national research committee and with the 1964 Helsinki declaration and its later amendments or comparable ethical standards. This study has obtained IRB approval from the ethics committee at Charité Campus Benjamin Franklin, Germany (application number EA4/002/18). All therapies were endorsed by an interdisciplinary tumor board and all patients who underwent their first TARE session between 2015 and 2022 were evaluated for inclusion. Exclusion criteria were missing baseline MRI in a timeframe > 60 days before TARE or missing follow up MRI ≥ 60 and ≤ 90 days afterwards and poor imaging quality (Fig 1).

### Y-90 radioembolization protocol

Board-certified interventional radiologists performed the interventions according to a standard protocol (S1 Protocol). At our institution, Y-90 radioembolization is performed with Yttrium 90 (Y-90) resin microspheres (SIR-Spheres®, Y-90 radioembolization Medical Limited, North Sydney, Australia). The prescribed activity of Y-90 resin microsphere was determined according to body surface area (BSA) method. In the cohort analyzed, TARE was performed as palliative treatment in sequential lobar fashion if feasible.

### Imaging technique

All patients underwent cross-sectional imaging before and after Y-90 radioembolization. CE MRI was performed with a 1.5 T (Siemens Avanto or Aera) imaging unit. A phased-array torso coil and 0.1 ml per kilogram body weight of intravenous dinatriumgadoxetat (Eovist/Primovist, Bayer Healthcare) were used. The MRI protocol included breath-hold unenhanced and CE T1-weighted 3D fat-suppressed spoiled gradient-echo imaging (section thickness, 2,5 mm; receiver bandwith, 64 kHz; flip angle, 10°) in the arterial phase (delay of 15 seconds after bolus tracking), portal venous phase (delay of 70 seconds), the delayed phase (delay 3 minutes) and the hepatobiliary excretion phase (delay of 20 minutes after administration). The arterial contrast phase was chosen for volumetric evaluation.

### Quantitative measurement of arterial tumor vascularization on CE MRI

Quantitative measurements of enhancement were performed with a semi-automated software (Philips IntelliSpace Portal V 8.0) and are given in milliliters. Accuracy and reproducibility were previously demonstrated [18,19]. A signal intensity greater two standard deviations than the average signal intensity measured within a region of interest (ROI) of 10 x 10 x 10 mm in the musculus erector spinae was defined an arterial vascularization. Before and after TARE, up to three corresponding lesions were analyzed and combined as surrogate total tumor volume (TTV) and enhancing tumor volume (ETV). The most representative lesions were selected based on size and reproducibility. Relative vascularization was calculated as the ratio of ETV and TTV, the segmentation methodology is illustrated in Fig 1.

### Statistical analysis

Statistical analyses were conducted with IBM SPSS STATISTICS, version 25 (IBM Corporation, Armonk, NY, USA) and R statistical software (version 2025, R Core Team, Vienna, Austria). Testing for normality was performed with the Shapiro Wilk test. Normally distributed, continuous data are presented with mean and standard deviation and compared with the two-sample t-test. Non-normally distributed data are expressed as median and interquartile range (IQR) and compared with the Mann–Whitney U test. We used maximally selected rank statistics (Wilcoxon-based) to determine optimal cutoffs for continuous imaging parameters with respect to continuous outcomes, followed by group comparisons using the Mann–Whitney U test, with all results interpreted as exploratory and hypothesis-generating. Overall survival is reported as median (mOS) with a 95% confidence interval, the impact of imaging and clinical baseline variables on mOS was analyzed with the Cox proportional hazard model, Kaplan-Meier curves and the log-rank test. Regression coefficients, hazard ratios (HR) and odds ratios (OR) as well as p-values are presented, whereas p-values < 0.05 are reported as significant. As this is an explorative study, no adjustment for multiple testing was applied.

## Results

### Patient characteristics

**Fig 2:**
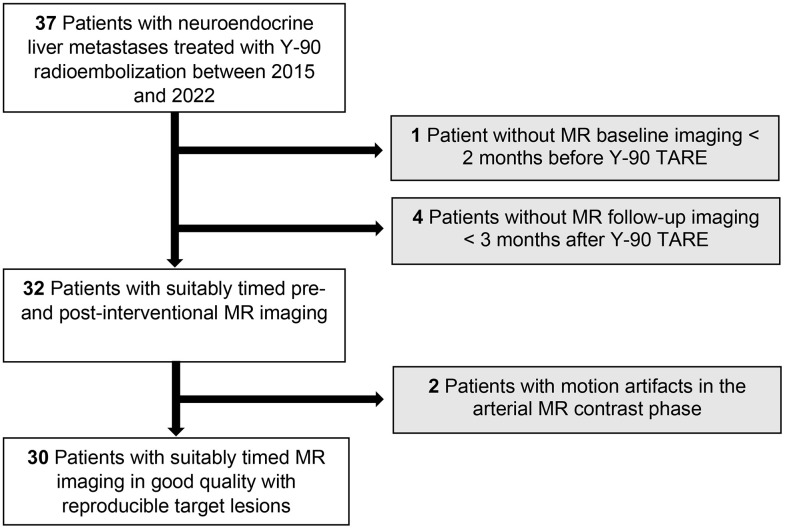
Inclusion criteria. Flowchart depicting included cases during the retrospective analysis.

Out of 37 patients treated for NELM with lobar TARE between 2015 and 2022, 7 patients were excluded due to missing or non-diagnostic MR caused by breathing artifacts in the arterial contrast phase (Fig 2). Excluded patients exhibited a significantly higher tumour burden and shorter overall survival (S1 Table). All included patients underwent baseline imaging within one or two days prior to TARE. Patient characteristics are summarized in Table 1. In total 24/30 patients (80%) with NELM underwent resection of their primary tumor which was in the upper gastrointestinal tract in 13/30 cases (43%). A total of 18/30 patients (60%) had prior liver resection, 50% of patients had extrahepatic metastases. Tumor grading was G2 in 16/30 patients (53%). All patients received systemic treatments prior to intraarterial therapy whereas 17/30 patients (57%) received no systemic treatment after TARE. In total 16/30 (53%) received PRRT. TARE was performed as sequential lobar treatment in 20/30 cases (66.7%).

**Table 1:** Patient Characteristics.

Demographics		
Number of Patients		30
Age in years, mean (SD)		63 (11)
Male Sex, n (%)		15 (50)
Body Mass Index in kg/m^2^, mean (SD)		25 (5)
Median Survival in months (95% CI) *		33.03 (21.32, 44.75)
Characteristics of the Tumor		
Grading, n (%)	G1	8 (27)
	G2	16 (53)
	G3	6 (20)
Primary Tumor, n (%)	Upper GI tract	13 (43)
	Lower GI tract	5 (17)
	Pancreas	5 (17)
	Kidney	2 (7)
	Lung	1 (3)
	Cervix	1 (3)
	Unknown	3 (10)
Portal Vein Thrombosis, n (%)	None	25 (83)
	Left	3 (10)
	Right	1 (3)
	Bilateral	1 (3)
Primary Tumor Resected, n (%)		24 (80)
Extrahepatic Metastases, n (%)		15 (50%)
KI-67 > 5%, n (%)		15 (50)
Tumor Volumes in ml, median (IQR)		327 (760)
Hepatic Tumour Burden in %, median (IQR)		17 (21)
Previous Surgical or Interventional Treatments		
Resection, n (%)		18 (60)
Transarterial Chemoembolisation, n (%)		1 (3.3)
Transarterial Embolization, n (%)		3 (10)
Ablation (Brachytherapy), n (%)		9 (30)
Systemic Therapies		
Before TARE only, n (%)		17 (57)
Before and after TARE, n (%)		13 (43)
Somatostation-Receptor-Directed Therapies, n (%)		24 (80)
Somatostatin Analoga, n (%)		20 (67)
Peptide Receptor Radionuclide Therapy, n (%)		16 (53)
Chemotherapy, n (%)		27 (90)
Tyrosin Kinase Inhibitors, n (%)		7 (23)
Everolimus, n (%)		16 (53)
Immunotherapy, n (%)		8 (27)
Specifics of radioembolization		
Sequential Lobar Therapy, n (%)		20 (66.7)
Hepatopulmonary Shunt in %, median (IQR)		2.80 (2.90)
Liver Volumes in ml, median (IQR)		1942 (1423.25)
Tumour Volumes in ml, median (IQR)		327.5 (760.75)
Activity in mBq, median (IQR)		1.8 (0.43)

*Kaplan-Meier estimator.

Abbreviations: CI: confidence interval, IQR: interquartile range, SD: standard deviation.

### MRI tumor segmentation at baseline and 3 months after treatment

**Fig 3:**
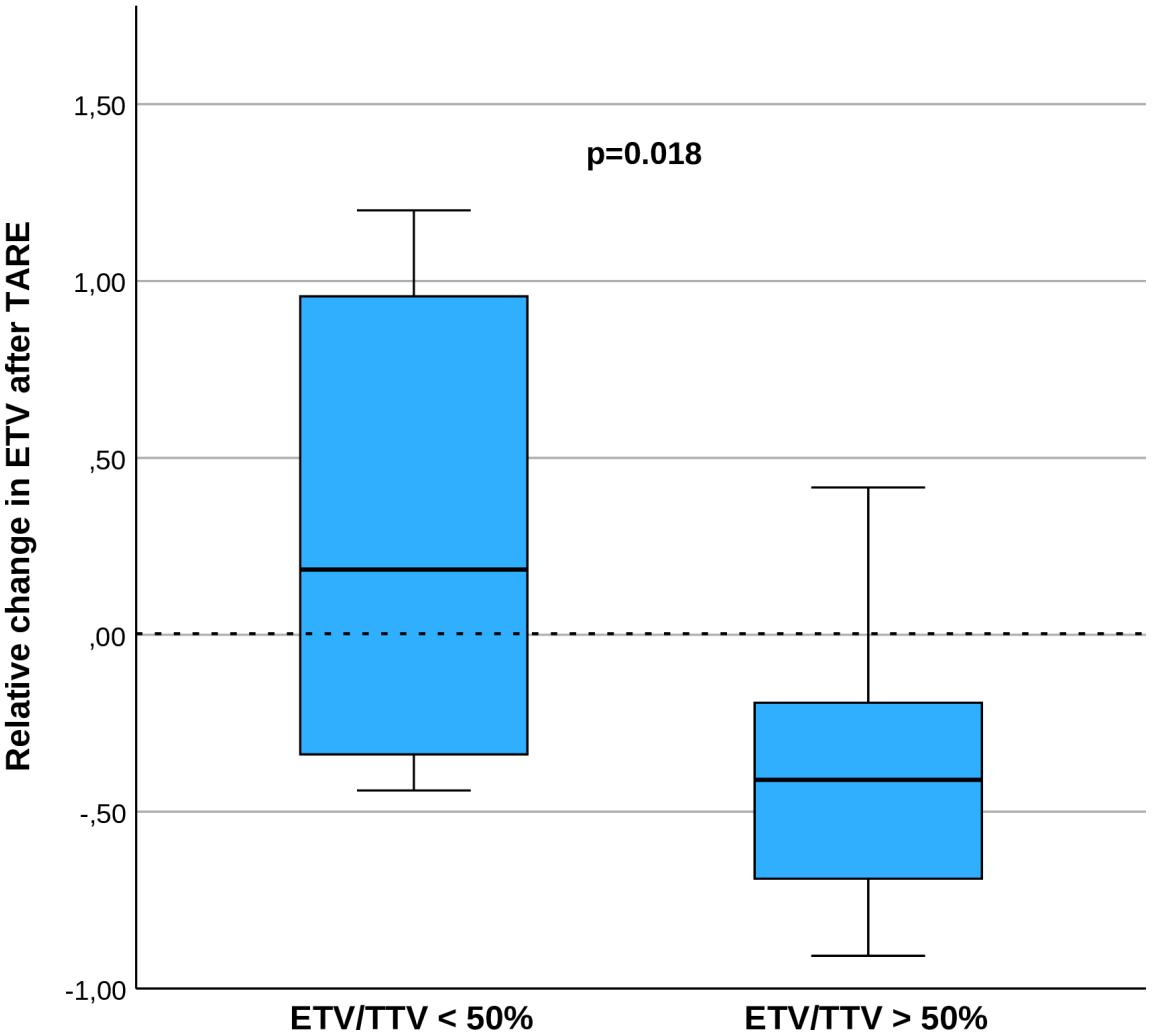
Impact of Baseline Vascularity on Cytoreduction after Radioembolization. Box plots illustrate the significantly different evolution of median enhancing tumor volume (ETV) from baseline to follow-up MRI after radioembolization depending on the baseline ratio of enhancing to total tumor volume (ETV/TTV) <or > 50%. A relative increase in ETV is illustrated with positive values above 0 (dotted line), a decrease below 0.

TTV and ETV on CE MRI both at baseline and three months after treatment were approximated by summarizing the volumes of a maximum of three dominant lesions in 30 patients (Table 2). Baseline median ETV and TTV were 77 ml (IQR 169 ml) and 151 ml (interquartile range, IQR 219 ml) respectively with a median ETV/TTV ratio of 78% (IQR 30%). Maximally selected Wilcoxon rank statistics were used to derive an exploratory cutoff of 42% for the baseline ETV/TTV ratio that maximized separation of ETV change on follow-up MRI; group differences were subsequently assessed using the Mann–Whitney U test. In accordance with recent evidence in HCC and with the aim of improving specificity, an ETV/TTV cutoff of 50% was applied in all subsequent analyses and was observed in 24 of 30 patients. In patients with a baseline ETV/TTV ratio >50%, the median relative decrease of ETV was 41% (IQR 51%) whereas patients with an ETV/TTV ratio <50% had a median increase in ETV of 18% with a larger IQR of 138% (p = 0.018; Table 2 and Fig 3). There was no significant difference in the relative change of ETV between the cohorts with or without prior PRRT.

**Table 2:** Tumor segmentation.

MRI Tumor Segmentation		Median (IQR)
TTV in ml	baseline	151 (219)
	follow-up	101 (140)
	Change in %	−27 (45)
ETV in ml	baseline	77 (169)
	follow-up	57 (133)
	change, %	−34 (61)
ETV/TTV ratio in %	baseline	78 (30)*
	follow-up	62 (52)
ETV change based on baseline vascularity**		
ETV/TTV < 50% in %		18 (138)
ETV/TTV > 50% in %		−41 (51)

* ETV/TTV ratio at baseline greater 50% in 24/30 cases.

** Significance reached p = 0.018 comparing ETV/TTV > 50% vs. < 50% and was assessed with the Mann–Whitney U test.

Abbreviations: ETV: enhancing tumour volume, IQR: interquartile range, ml: milliliter, TTV: total tumour volume.

### Predictors of patient survival

In univariable Cox regression (Table 3), prior resection and treatment with somatostatin analoga and PRRT had hazard ratios (HR) of 0.25 (p = 0.021) and 0.22 (0.024), respectively. Neither baseline demographic features, tumor characteristics nor different treatment regimens significantly affected patient survival. Considering tumor characteristics, grading or vascularization on baseline MRI were considered. Different treatment regimens included resection of the primary tumor, other systemic treatments such as immune checkpoint inhibition (ICI) or the TARE approach in terms of lobar or whole liver treatment. In patients who received systemic therapy exclusively before TARE (17/30), elevated baseline ETV/TTV, as continuous and dichotomized variable greater or lesser 50%, was associated with a HRs of 0.05 (p = 0.072) and 0.20 (p = 0.115) respectively. A tumor grading >2 and prior ablation of NELM with brachytherapy corresponded to HRs of 17.04 (p = 0.022) and 5.26 (p = 0.099), respectively.

**Table 3:** Univariable Cox regression analysis to estimate patient survival in NELM cohorts with or without additional therapy to TARE.

Univariable analysis	All patients		Systemic therapy			
			Pre-interventional		Pre- and post-interventional	
	n = 30		n = 17		n = 13	
Demographics	HR	p-value	HR	p-value	HR	p-value
Sex (male)	1.32	0.636	0.75	0.716	48.84	0.358
Age > 65 years	2.07	0.282	1.32	0.737	1.96	0.571
Characteristics of the Tumor						
Somatostatin Receptor Positivity	0.24	0.028	0.36	0.172	0.17	0.210
Tumor Grading > G2	2.15	0.261	17.04	0.022	1.76	0.646
Cancer of Unknown Primary	2.23	0.318	0.86	0.849	–	–
Primary Tumor Resected	1.21	0.857	2.68	0.362	–	–
Extrahepatic Metastases	2.40	0.162	0.80	0.763	4.96	0.175
KI-67 > 5%	1.49	0.496	2.46	0.230	0.57	0.632
Tumour burden	3.18	0.504	0.43	0.741	3.10	0.675
Portal Vein Thrombosis	1.07	0.930	1.92	0.454	0.04	0.558
Interventional or Surgical Treatments						
Liver Resection	0.25	0.021	0.72	0.657	0.21	0.121
Brachytherapy	1.01	0.983	5.26	0.099	1.00	1.000
Systemic Treatments						
Somatostatin Analoga	0.34	0.095	0.41	0.205	0.27	0.374
Peptide Receptor Radionuclide Therapy	0.22	0.024	0.30	0.145	0.28	0.265
Tyrosine Kinase Inhibitors	0.50	0.370	1.11	0.900	0.02	0.391
Immune Checkpoint Inhibition	1.64	0.427	1.21	0.799	1.36	0.793
Everolimus	1.48	0.527	2.85	0.333	0.20	0.175
Radioembolization Approach						
Sequential Lobar Therapy	1.37	0.621	0.57	0.440	2.37	0.459
Dose Reduction	0.28	0.099	0.51	0.535	0.39	0.415
Applied Dose (in mBq)	3.44	0.153	1.04	0.974	6.64	0.272
MRI Tumor Characterization						
TTV (in ml)	1.00	0.535	1.00	0.943	1.00	0.762
ETV (in ml)	1.00	0.727	1.00	0.715	1.00	0.719
ETV/TTV	0.71	0.737	0.05	0.072	2.07	0.524
ETV/TTV > 50%	0.95	0.937	0.20	0.115	2.11	0.524
Hepatopulmonary Shunt (in %)	1.06	0.462	1.01	0.951	1.10	0.719

Abbreviations: ETV: enhancing tumour volume, HR: hazard ratio, ml: milliliter, TTV: total tumour volume.

### Survival times with respect to type and timing of systemic therapy

The mOS of the whole cohort was 33.03 months (95% CI [21.32, 44.75]; Table 4). Patients treated with PRRT had the longest mOS of 60.8 months (no CI calculated due to the survival function not reaching 0.45) whereas patients receiving SSA therapy had a mOS of 33.03 months (95% CI [9.63,56.44]) and those not amenable to somatostatin-targeted therapy had a mOS of 6.97 months (95% CI [0.00, 14–63]) (p = 0.019). Patients who only received pre-interventional systemic therapy had an estimated mOS of 15.37 months (95% CI [4.64, 26.09]) whereas those who received both pre- and post-interventional systemic therapy had a mOS of 60.80 months which is most likely attributable to a survivorship bias (p = 0.020). In patients who only received pre-interventional systemic therapy, a baseline ETV/TTV ratio < 50% was associated with shorter mOS of 6.8 months compared to 15.37 months (95% CI [5.32, 25.41]) in patients with an ETV/TTV ratio > 50% (p = 0.082; Table 4).

**Table 4:** Survival with respect to type and timing of systemic therapy.

Cohort	mOS in months	95% CI	p-value
Whole Cohort	33.03	21.32 - 44.75	
Somatostatin-Directed Therapy			
No Somatostatin-Directed Therapy	6.97	0.00 - 14.63	0.019
Somatostatin Analoga Therapy	33.03	9.63 - 56.44	
Peptide Receptor Radionuclide Therapy	60.80	n.a.*	
Systemic Therapy			
Pre-interventional	15.37	4.64 - 26.09	0.020
Pre- and post-interventional	60.80	n.a.*	
Impact of baseline vascularity in patients with only pre-interventional systemic therapy			
ETV/TTV > 50%	15.37	5.32–25.41	0.082
ETV/TTV < 50%	6.80	n.a.**	

** not available due to lack of data points.

Abbreviations: CI: confidence interval, ETV: enhancing tumour volume, mOS: median overall survival, TARE: transarterial radioembolization, TTV: total tumour volume.

## Conclusion

This retrospective, single-center study in patients who underwent lobar TARE for NELM with BSA-based dosimetry, demonstrates arterial baseline vascularity >50% to be associated with a median ETV reduction of 41% after intraarterial therapy. In contrast, 20% of patients with poor baseline vascularity showed an 18% ETV increase (p = 0.018) on follow-up imaging. Eligibility for PRRT was the strongest predictor of survival with a HR of 0.22 (p = 0.024) corresponding to a mOS of 60.8 months, while prior PRRT did not significantly affect the change in ETV. In patients who received only pre-interventional systemic therapy, exploratory analyses suggested a potential association between higher baseline ETV/TTV ratios and prolonged survival (HR 0.05, p = 0.072).

Volumetric viability assessment on baseline MRI identified high- and low-vascularity cohorts that exhibited significant ETV reduction or continued growth following lobar TARE with standard dosing, consistent with prior findings in HCC [20]. The extent of ETV reduction was remarkably similar between hypervascular variants of both tumor entities, ranging around 41%. Hence, an ETV/TTV ratio of >50% may be a candidate cut-off for favorable tumor-to-liver dose distribution (tumor-to-liver ratio, TLR) and at least partial deterministic radiation damage. After validation in larger patient cohorts, such cut-off could help screen patient cohorts with a high probability of significant tumor debulking following conventional TARE. The efficacy of TARE does not seem to be affected by prior PRRT. With respect to the impact of baseline vascularity on patient survival, the sample size is too small and heterogenous to draw more definite conclusions. In a larger HCC cohort, however, time to target lesion progression was significantly longer in patients with high baseline vascularity and potential survival benefits were seen for combination therapy in these patients [17].

These findings align with current paradigms. As most gastroenteropancreatic NETs express somatostatin receptors, receptor-directed therapies have become the preferred treatment strategy whereas the roles of LDTs and surgery are to increase their efficacy as well as to control symptoms through debulking [21–23]. The NETTER-1 trial confirmed the efficacy of PRRT in controlling tumor growth and symptoms in advanced NETs who progressed on high-dose SSA therapy whereas the NETTER-2 trial proved the superiority of PRRT as first-line therapy which agrees with the survival outcomes we saw in our cohort [22,23]. Following lobar TARE, significant long-term liver toxicity with cirrhosis-like symptomatology was found in 7.6% of patients in the prospective RESiN registry. However, in recent retrospective analyses rates of 13% and 29% were reported and therefore TAE or TACE are favored for somatostatin receptor-avid disease in potential PRRT candidates [8,^24^-^25^–]. We found 20% of patients to exhibit unfavorable baseline vascularity with ETV/TTV ratios below 50% associated with continued post-interventional tumor growth probably due to poor TLRs, as suggested by prior correlation studies of vascularity and post-TARE single-photon emission computed tomography (SPECT) in HCC and NELM [15]. This percentage is in a comparable order of magnitude as the incidence of long-term toxicity following TARE in the literature and is not unexpected, as less tumoral uptake is invariably associated with increased radiation to the surrounding liver. This finding may explain to some extent why retrospective analyses found TARE inferior to TACE in terms of mOS, despite comparable local control rates [26]. One recent study using the same liver segmentation approach reported overall and enhancing tumor burden as strong prognostic factors of overall survival in patients with NELM undergoing intra-arterial therapies but did not delve deeper into the impact of baseline vascularization as predictor of TARE efficacy [27].

Our findings suggest that baseline vascularity warrants further investigation as a potential tool for both retrospective assessment of TARE efficacy and prospective response prediction. Secondary analyses of the NETTER-1 trial as well as a recent study on the impact of surgical resection linked higher tumor volumes to poorer PRRT response, supporting hepatic lesion debulking before treatment and thus fostering the role for adjunctive liver-directed therapy [23,28,29]. Although TARE potentially outperforms TACE and TAE at controlling unilobar disease as well as rapidly progressing diffuse liver involvement, there are reservations to its application in potential PRRT candidates due to ambiguous long-term safety data. The proposed baseline ETV/TTV ratio as an easily accessible imaging metric merits evaluation in existing, high-quality retrospective data such as the RESiN registry to potentially better understand risk factors of long-term radioembolization-induced chronic hepatotoxicity. Conversely, its potential utility for retrospective assessment and prospective prediction of TARE efficacy should be explored. There are different arguments why an increased utilization of TARE in NELM may be beneficial. First, in HCC patients who underwent TARE or TACE with complete radiological response, those treated with TARE were less likely to suffer local or general recurrence than those treated with TACE, adding to the body of evidence that TARE may be the superior LDT for local tumor control [30,31]. Similarly, patients with NELM and favorable baseline vascularity might benefit from TARE as first-line LDT both as segmentectomy for single lesions and as lobar treatment for multifocal disease. Second, a wider application of TARE in NELM offers potential synergisms with radiosensitizing chemotherapy such as the combination of capezitabine and temozolomide [32]. Third, well-differentiated neuroendocrine neoplasms generally have slow growth rates and low tumor mutation burden which is why the effectiveness of immune checkpoint inhibitors (ICIs) has been limited [33]. TARE was demonstrated to increase the susceptibility to ICI therapy in different solid tumors such as HCC or uveal melanoma and a pilot clinical study in patients with well-differentiated neuroendocrine neoplasms is currently underway to evaluate whether combining PDL-1 inhibitor pembrolizumab with LDT can result in abscopal effects [34–36].

The study has limitations that impact the generalizability of the findings. The cohort is small and mainly includes patients in whom TARE was used as a bailout strategy, in part explaining the unusually high proportion of patients who received chemotherapy or immunotherapy. Excluded patients exhibited a higher tumor burden and shorter overall survival, which limited the availability of follow-up imaging and may have introduced selection bias. Additional limitations include variability in the timing of baseline and follow-up imaging, the use of TARE as both whole-liver and sequential therapy, and the limited feasibility of meaningful analyses of time to radiologic progression and survival in patients receiving intensive pre- and post-interventional treatments. Moreover, the incidence of long-term radioembolization-induced chronic hepatotoxicity was not recorded, and SPECT Y-90 tumor absorbed-dose metrics were not performed to elucidate the relationship between baseline vascularity and absorbed doses.

To conclude, we demonstrate an association of baseline vascularity and significant radiation-induced necrosis after conventional TARE of NELM. An ETV/TTV ratio >50% allowed to identify patients that significantly reduced their ETV. About 20% of patients with poor baseline vascularity increased their ETV probably due to an unfavorable TLR which, in turn might help identify cohorts at risk for long-term radioembolization-induced chronic hepatotoxicity. This might position the baseline ETV/TTV ratio as a novel metric for the retrospective analysis of high-quality datasets on treatment efficacy and toxicity, and as a potential—yet to be validated—tool to identify patients who may benefit from TARE for cytoreduction, either alone, in combination with radiosensitizing chemotherapy, or as an adjunct to ICI therapy.

## Supporting information

S1 Protocol Institutional protocol for Y-90 radioembolization. (DOCX)

S1 Table Characteristics of included and excluded patients. (DOCX)

S1 Data Raw volumetric measurements, confounding variables, survival data. (XLSX)

## Abbreviations

CE: contrast enhanced
BSA: body surface area
CRLM: colorectal liver metastases
ETV: enhancing tumor volume
HCC: hepatocellular carcinoma
HR: hazard ratio
ICI: immune checkpoint inhibitor
IQR: interquartile range
LDT: liver-directed-therapies
mOS: median overall survival
NELM: neuroendocrine liver metastases
OS: overall survival
PCC: Pearson correlation coefficient
PRRT: peptide receptor radionuclide therapy
qEASL: quantitative European Association for the Study of the Liver (criteria)
ROC: receiver operating characteristic
SPECT: single-photon emission computed tomography
TACE: transarterial chemoembolization
TAE: transarterial embolization
TARE: transarterial radioembolization
TLR: tumor-to-liver ratio
TTV: total tumor volume
Y-90: Yttrium-90(-radioembolization)
