# Supplementary material for: Yttrium-90 radioembolization for neuroendocrine liver metastases: baseline vascularity predicts cytoreduction efficacy
Source: PLoS One. 2026 Jul 14;21(7):e0352018. doi: 10.1371/journal.pone.0352018 (PMC13367693; doi:10.1371/journal.pone.0352018)
Supplement: S1 Table — (DOCX) [file pone.0352018.s002.docx]

**Supplement Table 1: Characteristics of included and excluded patients**

| Patient Demographics | | Included | Excluded | Sign. |
| --- | --- | --- | --- | --- |
| Number of Patients | | 30 | 7 |  |
| Age in years, mean (SD) | | 63 (11) | 69 (13) | 0.265 |
| Male Sex, n (%) | | 15 (50) | 3 (43) | 1.000 |
| Median Survival in months, (95% CI) * | | 33.03  (21.32, 44.75) | 7.03  (3.90, 10.15) | 0.011 |
| Characteristics of the Tumor | | |  |  |
| Grading >2, n (%) | G1 | 6 (20) | 1 (14.3) | 1.000 |
| Primary Tumor, n (%) | GI tract | 18 (60) | 2 (29) |  |
|  | Pancreas | 5 (17) | 0 |  |
|  | Kidney | 2 (7) | 0 |  |
|  | Lung | 1 (3) | 3 (43) |  |
|  | Cervix | 1 (3) | 0 |  |
|  | Unknown | 3 (10) | 2 (29) |  |
| PVT, n (%) | None | 25 (83) | 7 |  |
|  | Left | 3 (10) | 0 |  |
|  | Right | 1 (3) | 0 |  |
|  | Bilateral | 1 (3) | 0 |  |
| Primary Tumor Resected, n (%) | | 24 (80) |  |  |
| Extrahepatic Metastases, n (%) | | 15 (50%) | 3 (43) | 1.000 |
| KI-67>5%, n (%) | | 15 (50) | 3 (43) | 1.000 |
| Hepatic Tumour Burden in %, median (IQR) | | 17 (21) | 43 (19) | 0.010 |
| Previous Surgical or Interventional Treatments | | |  |  |
| Resection, n (%) | | 18 (60) | 3 (43) | 0.437 |
| Transarterial Chemoembolisation, n (%) | | 1 (3.3) | 0 |  |
| Transarterial Embolization, n (%) | | 3 (10) | 3 | 0.019 |
| Ablation (Brachytherapy), n (%) | | 9 (30) | 2 (29) | 1.000 |
| Systemic Therapies | | |  |  |
| Before TARE only, n (%) | | 17 (57) | 4 (57) | 1.000 |
| Before and after TARE, n (%) | | 13 (43) | 3 (43) | 1.000 |
| Somatostation-Receptor-Directed Therapies, n (%) | | 24 (80) | 6 (86) | 1.000 |
| Somatostatin Analoga, n (%) | | 20 (67) | 6 (86) | 0.649 |
| Peptide Receptor Radionuclide Therapy, n (%) | | 16 (53) | 4 (57) | 1.000 |
| Chemotherapy, n (%) | | 27 (90) | 2 (29) | 0.002 |
| Tyrosin Kinase Inhibitors, n (%) | | 7 (23) | 0 | 0.306 |
| Everolimus, n (%) | | 16 (53) | 3 (43) | 0.693 |
| Immunotherapy, n (%) | | 8 (27) | 0 | 0.308 |
| Specifics of radioembolization | | |  |  |
| Sequential Lobar Therapy, n (%) | | 20(66.7) | 5 (71) | 1.000 |
| Hepatopulmonary Shunt in %, median (IQR) | | 2.80 (2.90) | 2.10 (2.90) | 0.977 |
| Liver Volumes in ml, median (IQR) | | 1942 (1423.25) | 2710 (1675.00) | 0.186 |
| Tumour Volumes in ml, median (IQR) | | 327.5 (760.75) | 944 (1014.00) | 0.059 |
| Activity in mBq, median (IQR) | | 1.8 (0.43) | 1.8 (0.71) | 0.338 |
| *Kaplan-Meier estimator  Abbreviations: CI: confidence interval, IQR: interquartile range, PVT: portal vein thrombosis, SD: standard deviation | | | | |
